# Retention and longitudinal change in Insight 46, an intensive neuroscience sub-study of the 1946 British birth cohort

**DOI:** 10.1186/s13104-025-07323-y

**Published:** 2026-01-22

**Authors:** Sarah E. Keuss, Kirsty Lu, Sarah-Naomi James, Jennifer M. Nicholas, William Coath, Ashvini Keshavan, David M. Cash, Carole H. Sudre, Josephine Barnes, Heidi Murray-Smith, Andrew Wong, Rebecca Street, Marcus Richards, Jonathan M. Schott

**Affiliations:** 1 Dementia Research Centre, Department of Neurodegenerative Disease, UCL Queen Square Institute of Neurology, University College London, 8-11 Queen Square, London, WC1N 3BG UK; 2 MRC Unit for Lifelong Health and Ageing at UCL, University College London, London, UK; 3 Department of Medical Statistics, London School of Hygiene and Tropical Medicine, London, UK; 4 UK Dementia Research Institute at UCL, University College London, London, UK; 5 Hawkes Institute, Department of Computer Science, University College London, London, UK

**Keywords:** Retention, Attrition, Population, Birth cohort, Longitudinal, Neuroscience, Ageing

## Abstract

**Objectives:**

Participant retention is a significant challenge in ageing and dementia research. This analysis investigated (a) factors associated with retention in Insight 46, a neuroscience sub-study of the 1946 British birth cohort, and (b) clinical and cognitive changes over 2.5 years of follow-up.

**Results:**

Of 502 participants assessed at baseline (mean[SD] age: 70.5[0.7] years), 442 returned for follow-up (mean[SD] interval: 2.5[0.3] years), representing a retention rate of 88%. Being β-amyloid positive (measures using positron emission tomography), female sex, and older age at baseline associated with lower odds of retention, while completion of neuroimaging and better cognitive performance at baseline– particularly on memory testing– related to higher odds of retention. By the time of follow-up, 14 participants were deceased, 12 of whom were female. Over follow-up, improvements were noted in certain cognitive tests (face-name test, logical memory delayed recall) with declines seen in others (mini-mental state examination, digit-symbol substitution test). Increases in self- and informant-reported cognitive complaints, cognitive disorder diagnoses, and motor abnormalities were also observed, alongside declines in blood pressure. These results have implications for the interpretation and generalisability of Insight 46 data and may be relevant to the planning of other longitudinal studies in this field.

**Supplementary Information:**

The online version contains supplementary material available at 10.1186/s13104-025-07323-y.

## Introduction

Longitudinal studies of older adults contribute to our understanding of ageing and dementia. Participant retention poses a significant challenge for these studies and may influence the interpretation and generalisability of their results. Insight 46 is a prospective longitudinal sub-study of the MRC National Survey of Health and Development (NSHD; 1946 British birth cohort) which aims to investigate influences on brain health in later life [1, 2]. NSHD members recruited to Insight 46 have previously been shown to have higher self-rated health, education, and socioeconomic position than those who were not [3]. Extending this work, the current analysis aimed to examine factors associated with retention at follow-up. A further aim was to assess clinical and cognitive changes over a 2.5-year interval.

## Methods

### Study protocol and recruitment

Study protocols are detailed elsewhere [1, 3]. NSHD members were eligible to take part in Insight 46 if they had specific life course data available, had previously expressed a willingness to travel to London, and had no contraindications to magnetic resonance imaging (MRI) or positron emission tomography (PET). An invitation was sent to participants by post and then participants were contacted by telephone if interested. 502 NSHD members meeting eligibility criteria were recruited to attend University College London for clinical and cognitive assessment and multi-modal neuroimaging at two time points. Baseline visits were completed between May 2015 and January 2018. Follow-up initially ran from January 2018 until March 2020, when the study was interrupted by the coronavirus pandemic. It resumed with a combination of in-person and remote visits in August 2020 and finished in January 2021. The interval between visits was intended to be two years to allow adequate time for change to become detectable, and since this might be considered an appropriate length for a putative clinical trial. The actual interval was slightly longer than this, mostly due to scheduling delays during the coronavirus pandemic.

### Measures

Similar to a previous analysis of predictors of recruitment to Insight 46 [3], socioeconomic and health-related life course measures were ascertained from NSHD data and categorised into groups. In order to facilitate meaningful analysis, some measures were operationalised slightly differently due to smaller participant numbers. In addition to NSHD data, Insight 46 measures were used. A small number of Insight 46 participants (*n* = 29) completed a remote visit at follow-up due to the coronavirus pandemic. For these participants, the study protocol was adapted so that it could be performed by telephone or video call. Otherwise, the same protocol was used for data collection at both time points.

#### NSHD data

Childhood cognition was measured from four tests of verbal and non-verbal memory [4]. Education level by age 26 was categorised into: advanced (A-level) or equivalent or a higher degree; ordinary (O-level) or equivalent or vocational qualifications; or none. Socioeconomic position (SEP) at age 53 was grouped into manual or non-manual occupations. Overall disease burden at age 69 was grouped into ≥ 3, 2, 1 or no clinical disorders [5]. Self-rated participant health was obtained at age 68 and categorised as excellent, very good, good, or fair/poor. An office-based Framingham Heart Study Cardiovascular Risk Score (FHS-CVS) was derived at age 69 [6], and categorised into low (< 10%), intermediate (10–20%) or high risk (> 20%) of developing cardiovascular disease within the next ten years, based on established cut-offs. APOE ε4 status (carrier or non-carrier) was determined previously [7]. 

#### Insight 46 data

Blood pressure was calculated as the average of two readings, the first measured after three minutes in the supine position, and the second after a further one minute. Body mass index was calculated as weight in kilograms divided by height in metres squared. Parkinsonian signs were assessed using the Unified Parkinson’s Disease Rating Scale (UPDRS) Part III [8]. Cognitive tests included the mini-mental state examination (MMSE) [9], digit symbol substitution test (DSST) from the Wechsler Adult Intelligence Scale-Revised [10], logical memory delayed recall from the Wechsler Memory Scale-Revised [11], and face-name test (FNAME-12) [12]. A preclinical Alzheimer’s cognitive composite (PACC) was generated, representing an average of MMSE, DSST, logical memory delayed recall and FNAME-12 z-scores (based on the performance of the full Insight 46 sample at baseline) [13, 14]. Dementia, mild cognitive impairment (MCI), and major brain disorder were determined as reported elsewhere [13]. Subjective cognitive decline was measured using the SCD-Q part I MyCog questionnaire [15]. Informant history was obtained using the AD8 [16]. Anxiety was examined using the state and trait anxiety inventory [17]. β-amyloid (Aβ) status was determined from partial volume corrected Florbetapir PET standardised uptake value ratios, with a whole cerebellum reference [18]. White matter hyperintensity volume (WMHV) was measured from T1-weighted and fluid-attenuated inversion recovery (FLAIR) MRI using Bayesian model selection (BaMoS) [19]. Whole brain, hippocampal and total intracranial volumes (TIV) were segmented from T1-weighted MRI using automated techniques [20–22]. 

### Statistical analysis

All analyses were performed in Stata 18.

#### Association of measures with retention

Logistic regression was used to assess associations of measures with retention (returner versus non-returner status). Models were initially unadjusted (except for models with volumetric imaging measures as predictors which were adjusted for TIV), and then adjusted for baseline age, sex, childhood cognition, education level, and SEP. If measures were found to have significant associations with retention, they were then examined together as predictors in a single model to determine their independent effects. Sensitivity analyses were also performed (i) excluding participants with dementia or MCI at baseline; and (ii) excluding participants who were deceased by time of follow-up.

#### Differences in measures between baseline and follow-up

Differences in continuous Insight 46 measures between baseline and follow-up were tested using a paired t-test (or a Wilcoxon signed-rank test if non-normally distributed), and differences in categorical Insight 46 measures were tested using a McNemar test. Differences were assessed in cognitive and clinical data only since longitudinal analysis of neuroimaging data involves additional considerations which are beyond the scope of this study. Sensitivity analyses were also performed excluding participants who completed a remote visit at follow-up since this may have influenced the results.

## Results

Of 502 participants assessed at baseline (mean[SD] age: 70.5[0.7] years), 442 returned for follow-up (mean[SD] interval: 2.5[0.3] years). The remaining 60 were either deceased (*n* = 14) or no longer able or willing to take part (*n* = 46). Thus, the retention rate was 88% overall and 91% among participants still alive. Reasons for dropout are summarised in Table 1.

**Table 1:** Primary reasons reported by participants for dropout

Primary reason	Number of participants
Unable or unwilling to have a remote visit during the pandemic	15
Health issues	8
Not wanting to travel to London again	6
Carer responsibilities	5
Worries about study tests or incidental findings	4
Being unable or unwilling to undergo neuroimaging	2
No reason given	6

### Measures associated with retention

Older age at baseline and female sex were associated with lower odds of retention, such that non-returners were six months older on average than returners, and 85% of females returned for follow-up versus 91% of males (Table 2). Being Aβ positive at baseline also related to lower odds of retention, with 84% of Aβ positive participants returning for follow-up compared to 91% of Aβ negative participants (Table 2). Completion of neuroimaging at baseline was associated with higher odds of retention, such that 95% of scanned participants returned for follow-up compared to 83% of participants who did not complete a scan (Table 2). Better cognitive performance at baseline– particularly on memory testing– also related to higher odds of retention, while being classified as having dementia or MCI at baseline related to lower odds of retention, although statistical power for this finding was low since only 2.8% (*n* = 14) met these criteria (Table 2). Effects of baseline cognition were strengthened after adjustment for age, sex, childhood cognition, education level, and SEP (Table 2). There were no consistent associations between retention and socioeconomic or health-related life course measures, although there was a non-significant trend towards higher retention in those with excellent self-rated health at age 68 (Table 2).

**Table 2:** Associations of demographic, life course, and baseline Insight 46 measures with participant retention (max. n = 502)

Measure		Returners (max. *n* = 442)	Non-returners (max. *n* = 60)	Returner vs. non-returner status: unadjusted results			Returner vs. non-returner status: adjusted results†		
				OR	95% CI	*p*	OR	95% CI	*p*
**Demographic and life course**									
Age at baseline Insight 46 visit, years, mean (SD)		70.6 (0.7)	71.1 (0.6)	0.32	0.20, 0.52	< 0.01*	0.33	0.20, 0.53	< 0.01*
Female sex (versus male), n (%)		209 (47.3)	37 (61.7)	0.56	0.32, 0.97	0.04*	0.50	0.28, 0.89	0.02*
Education level	Advanced (versus none), n (%)	233 (52.7)	40 (66.7)	0.76	0.35, 1.64	0.09	0.72	0.28, 1.82	0.12
	Ordinary (versus none), n (%)	140 (31.7)	11 (18.3)	1.66	0.66, 4.19		1.57	0.58, 4.26	
SEP, non-manual job (versus manual), n (%)		376 (84.6)	52 (86.7)	0.85	0.38, 1.86	0.68	0.87	0.37, 2.09	0.76
Childhood cognition, z-score, mean (SD)		0.38 (0.73)	0.44 (0.80)	0.91	0.63, 1.31	0.61	1.10	0.71, 1.72	0.66
APOE ε4 carrier (non-carrier as reference), n (%)		129 (29.3) *n* = 440^a^	19 (31.7)	0.90	0.50, 1.60	0.71	0.78	0.43, 1.44	0.44
Disease burden at age 69	≥ 3 (versus none), n (%)	67 (15.3)	10 (17.5)	0.91	0.39, 2.12	0.94	0.85	0.35, 2.06	0.92
	2 (versus none), n (%)	89 (20.4)	10 (17.5)	1.21	0.52, 2.79		1.18	0.49, 2.80	
	1 (versus none), n (%)	163 (37.3)	21 (36.8)	1.05	0.53, 2.10		0.92	0.45, 1.90	
		*n* = 437^a^	*n* = 57^a^						
Self-rated health at age 68	Excellent (versus fair/poor), n (%)	61 (14.2)	4 (7.3)	1.85	0.43, 7.87	0.53	2.24	0.50, 10.09	0.64
	Very good (versus fair/poor), n (%)	217 (50.6)	18 (32.7)	0.91	0.30, 2.75		1.17	0.37, 3.71	
	Good (versus fair/poor), n (%)	118 (27.5)	29 (52.7)	0.79	0.25, 2.51		1.08	0.32, 3.56	
		*n* = 429^a^	*n* = 55^a^						
FHS Cardiovascular Risk Score at age 69	High (versus low), n (%)	261 (60.3)	33 (60.0)	1.10	0.44, 2.79	0.97	0.56	0.18, 1.74	0.19
	Intermediate (versus low), n (%)	129 (29.8)	16 (29.1)	1.13	0.41, 3.06		1.24	0.44, 3.55	
		*n* = 433^a^	*n* = 55^a^						
**Baseline Insight 46**									
UPDRS, score out of 52, median (IQR)		3 (1–6)	3 (2–6) *n* = 56^a^	0.98	0.92, 1.04	0.46	0.99	0.93, 1.06	0.84
Informant concern, AD8 ≥ 2 (< 2 as reference), n (%)		23 (5.2)	3 (5.0)	1.04	0.30, 3.58	0.95	1.05	0.29, 3.76	0.94
Dementia or MCI (none as reference), n (%)		10 (2.3)	4 (6.7)	0.32	0.10, 1.07	0.06	0.36	0.10, 1.30	0.12
Major brain disorder (none as reference), n (%)		38 (8.6)	4 (6.7)	1.32	0.45, 3.83	0.61	1.17	0.39, 3.49	0.78
Subjective decline, MyCog score out of 20, median (IQR)		4 (2–7)	3 (1–6)	1.03	0.95, 1.11	0.47	1.03	0.95, 1.11	0.46
Anxiety levels	State score out of 80, median (IQR)	28 (23–34)	30 (25–40)	0.97	0.94, 1.00	0.08	0.98	0.95, 1.02	0.36
	Trait score out of 80, median (IQR)	30 (26–36)	32 (26–39)	0.99	0.95, 1.02	0.36	0.99	0.96, 1.03	0.73
MMSE, score out of 30, median (IQR)		30 (29–30)	30 (29–30)	1.07	0.83, 1.38	0.58	1.10	0.84, 1.43	0.48
DSST, score out of 93, mean (SD)		47.8 (10.4) *n* = 441^a^	46.4 (10.3)	1.01	0.99, 1.04	0.34	1.02	0.99, 1.05	0.13
LMDR, score out of 25, mean (SD)		11.6 (3.7)	10.6 (3.6)	1.08	1.00, 1.16	0.05*	1.14	1.05, 1.24	< 0.01*
FNAME-12, score out of 96, mean (SD)		65.5 (18.1) *n* = 440^a^	63.3 (19.7)	1.01	0.99, 1.02	0.37	1.02	1.00, 1.04	0.04*
PACC, z-score, mean (SD)		0.02 (0.72)	-0.13 (0.79)	1.30	0.92, 1.84	0.14	1.66	1.11, 2.49	0.01*
Completion of neuroimaging (non-completion as reference), n (%)		421 (95.3)	50 (83.3)	4.01	1.79, 9.00	< 0.01*	4.05	1.78, 9.18	< 0.01*
Aβ + PET status (Aβ- as reference), n (%)		92 (22.3) *n* = 413^a^	18 (36.7) *n* = 49^a^	0.49	0.26, 0.92	0.03*	0.46	0.23, 0.89	0.02*
WMHV on MRI, ml, median (IQR)		3.1 (1.6–6.8) *n* = 408^a^	3.0 (1.7–5.8) *n* = 47^a^	1.00	0.95, 1.05	0.95	1.02	0.95, 1.08	0.61
Whole brain volume on MRI, ml, mean (SD)		1104 (98) *n* = 418^a^	1067 (105) *n* = 50^a^	1.01	1.00, 1.01	0.03*	1.01	1.00, 1.01	0.08
Total hippocampal volume on MRI, ml, mean (SD)		6.3 (0.7) *n* = 418^a^	6.2 (0.7) *n* = 50^a^	1.13	0.68, 1.89	0.63	1.02	0.60, 1.72	0.94

Abbreviations: SD = standard deviation; IQR = interquartile range; SEP = socioeconomic position; UPDRS = unified Parkinson’s disease rating scale; MCI = mild cognitive impairment; MMSE = mini-mental state examination; DSST = digit symbol substitution test; LMDR = logical memory delayed recall; FNAME-12 = face-name test; PACC = preclinical Alzheimer cognitive composite; APOE = apolipoprotein E; Aβ = β-amyloid; PET = positron emission tomography; WMHV = white matter hyperintensity volume; MRI = magnetic resonance imaging. †adjusted for age at baseline Insight 46 visit, sex, childhood cognition, education, socioeconomic position. *significant at *p* ≤ 0.05. ^a^number of participants with available data if below maximum possible

Effects of measures associated with retention were not meaningfully altered when examined together as predictors in a combined model (Additional File 1: Table S1 and Table S2). Results were broadly similar after excluding participants with dementia or MCI at baseline (Additional File 1: Table S3). After excluding deceased participants (Additional File 1: Table S4), the effect of sex was attenuated. Further analysis revealed that females were more likely to have died by time of follow-up than males (χ^2^ = 7.7664; *p* < 0.01), with females accounting for 12 out of 14 deceased participants.

### Differences in measures between baseline and follow-up

There were significant differences in most measures between time points (Table 3). There was a mixed picture for cognitive measures, with significantly better FNAME-12 and logical memory delayed recall performance on average at follow-up, but significantly poorer DSST, MMSE and PACC performance on average, and subjectively worse cognition (Table 3). The proportion of participants with dementia or MCI increased by 3.4% points at follow-up, while informant-reported cognitive concerns increased by 12.9% points (Table 3). Mean UPDRS scores also increased slightly, while mean blood pressure decreased, and mean body mass index and general anxiety remained the same (Table 3). Results were similar after excluding participants who were assessed remotely (Additional File 1: Table S5).

**Table 3:** Differences in clinical and cognitive Insight 46 measures between baseline and follow-up (max. *n* = 442)

Measure		Baseline	Follow-up	Percentage point or mean (SD) change	*p*-value
UPDRS, score out of 52, median (IQR)		3 (1–6)	5 (3–8) *n* = 431^a^	2.6 (4.4) *n* = 431^a^	< 0.01*
Supine blood pressure	Systolic, mmHg, mean (SD)	138.5 (17.0) *n* = 441^a^	136.2 (17.2) *n* = 412^a^	-2.1 (17.8) *n* = 411^a^	0.02*
	Diastolic, mmHg, mean (SD)	74.2 (9.9) *n* = 441^a^	72.3 (9.3) *n* = 412^a^	-1.8 (10.3) *n* = 411^a^	< 0.01*
Body mass index, kg/m^2^, mean (SD)		27.7 (4.5)	27.6 (4.3) *n* = 414^a^	-0.1 (1.6) *n* = 414^a^	0.33
Anxiety levels	State score out of 80, median (IQR)	28 (23–34)	27 (23–33) *n* = 437^a^	-1.0 (6.8) *n* = 437^a^	0.02*
	Trait score out of 80, median (IQR)	30 (26–36)	30 (26–36) *n* = 439^a^	-0.1 (5.6) *n* = 439^a^	0.96
Informant concern, AD8 ≥ 2, n (%)		23 (5.2)	80 (18.1)	12.9	< 0.01*
Dementia or MCI, n (%)		10 (2.3)	25 (5.7)	3.4	< 0.01*
Major brain disorder, n (%)		38 (8.6)	43 (9.7)	1.1	0.06
Subjective decline, MyCog score out of 20, median (IQR)		4 (2–7)	4 (1–8)	0.5 (3.1)	< 0.01*
MMSE, score out of 30, median (IQR)		30 (29–30)	29 (28–30) *n* = 413^a^	-0.30 (1.21) *n* = 413^a^	< 0.01*
DSST, score out of 93, mean (SD)		47.8 (10.4) *n* = 441^a^	46.0 (10.8) *n* = 440^a^	-1.85 (5.38) *n* = 440^a^	< 0.01*
LMDR, score out of 25, mean (SD)		11.6 (3.7)	12.3 (3.6)	0.66 (3.18)	< 0.01*
FNAME-12, score out of 96, mean (SD)		65.5 (18.1) *n* = 440^a^	66.5 (19.4) *n* = 432^a^	1.05 (10.27) *n* = 430^a^	0.01*
PACC, z-score, mean (SD)		0.02 (0.72)	-0.03 (0.81)	-0.06 (0.44)	< 0.01*

Abbreviations: SD = standard deviation; IQR = interquartile range; UPDRS = unified Parkinson’s disease rating scale; MCI = mild cognitive impairment; MMSE = mini-mental state examination; DSST = digit symbol substitution test; LMDR = logical memory delayed recall; FNAME-12 = face-name test; PACC = preclinical Alzheimer cognitive composite

*significant at *p* ≤ 0.05 ^a^number of participants with available data if below maximum possible

### Post-hoc analyses

Post-hoc analyses were performed using inverse probability weighting to adjust for attrition (Additional File 1: Table S6– see Table legend for methods). Changes in cognition between baseline and follow-up were not meaningfully altered in weighted compared to unweighted analyses (Additional File 1: Table S6). In both weighted and unweighted analyses, rates of change in cognition did not differ significantly between Aβ positive and negative participants (Additional File 1: Table S6).

## Discussion

Rates of retention in population-based studies of older adults vary in the literature, ranging from 55 to 95%, [23] but the rate in Insight 46–88% overall and 91% among participants still alive– is comparatively high. This is despite follow-up being disrupted by the coronavirus pandemic and may reflect that Insight 46 is a somewhat selective sample, having been recruited based on the availability of relevant life course data (and hence high levels of prior involvement in the NSHD) [1, 24]. The NSHD has also been running continuously since its inception and keeps in regular contact with members through birthday cards and updates, as well as responding to individual questions and feedback from members, and involving them in the design of study assessments and dissemination of findings [24]. 

While retention in Insight 46 was high, several factors were found to be independently related to retention. Notably, being Aβ positive was associated with lower odds of retention, with 16% of Aβ positive participants not returning for follow-up. The reason for this finding is unclear since participants were not informed of their Aβ status owing to ethical reasons. Previous Insight 46 analyses have demonstrated greater levels of subjective cognitive complaints and anxiety among Aβ positive participants [25]. Thus, it may be that they were more likely to drop out due to concerns about their performance or feeling less able to cope with the demands of the study. Insight 46 participants recently underwent remote cognitive testing, which may be associated with less anxiety, and it could be informative to assess whether this facilitated participation among Aβ positive participants.

Interestingly, females were less likely to return for follow-up than males. Sex differences in retention have been reported elsewhere, though not consistently [26, 27]. In Insight 46, 12 out of the 14 participants who had died by time of follow-up were female, as were 4 out of 5 participants who did not return due to caring responsibilities. While the latter finding is consistent with the traditional role of females as caregivers, the higher mortality in females is somewhat unexpected since women tend to live longer than men. Examining death certificate data, cancer was the main cause of death among females (*n* = 8).

Despite participants being virtually identical in age, non-returners were slightly older on average at the time of their baseline visit than returners. Whether this represents a genuine age effect is unclear. Since age at visit was determined by the order of recruitment and the length of time taken to complete data collection, it may be that participants seen earlier in the study were healthier or more motivated to take part. Another consideration is the coronavirus pandemic which led to scheduling delays and drop-out among those invited for follow-up towards the end of data collection. Disentangling the impact of the pandemic is not straightforward; however, there remained significant differences in retention by age– and by sex, Aβ status, and cognition– after excluding participants whose follow-up was delayed or did not happen specifically because of the pandemic (data not shown).

Consistent with other longitudinal studies in this field [23], better cognition was related to higher retention. Participants who completed neuroimaging at baseline were also more likely to return, perhaps because participants who did not manage a scan at baseline felt discouraged from returning due to their previous experience– most suffered claustrophobia– or because they felt that ongoing participation was not worthwhile without a scan, though only two participants disclosed this as a specific reason for dropout.

Most measures showed significant, albeit small, differences between time points. While caution should be taken not to overinterpret small changes, it is interesting that FNAME-12 and logical memory delayed recall scores improved significantly on average, since both tests involve learning and recall of new information, making them more susceptible to practice effects [28, 29]. On the other hand, performance on the other PACC components– the MMSE and DSST– both declined on average and therefore must be driving the small decrease observed in mean PACC score. This is consistent with the DSST being highly sensitive to small changes in cognition over time, likely because it assesses processing speed [10]. The magnitude of decline on the DSST (equivalent to -0.7 points per year) is consistent with other reports of the association between older age and slower performance on this test [30]. The decline on the MMSE may be influenced by a ceiling effect, since scores were generally high at baseline and most participants could only get worse or remain stable [31]. 

Rates of change in cognition did not differ significantly by Aβ status, unlike in some other preclinical cohorts [32], perhaps reflecting the short follow-up and that Aβ positive individuals at this age are mostly expected to be at an early stage on the Alzheimer’s disease continuum [18]. Notably, these comparisons were unchanged in the weighted analysis, suggesting that participant drop-out may not be causing much bias when comparing Aβ positive and negative participants. Future work will examine the effects of other factors on rates of change in cognition, with a view to better understanding the factors the determinants of cognitive decline (and also preserved cognitive function) in later life.

### Limitations

Insight 46 participants are all white, and findings may differ in other racial or ethnic groups. They were also recruited from a birth cohort and assessed at a single centre in the UK, so results may not be translatable to studies with other recruitment methods or those conducted across multiple centres or different geographic areas.

## Conclusion

In summary, the current analysis highlights that high levels of participant retention can be achieved in an intensive neuroscience study of a population-based elderly cohort. Aβ positive participants and those with poorer cognition were less likely to return for follow-up, as were female participants and those who were older. Further research is needed to examine the relationship between Aβ status and participant retention.

## Electronic supplementary material

Below is the link to the electronic supplementary material.

Supplementary Material 1

## Data Availability

Data from the NSHD are curated and stored by the MRC Unit for Lifelong Health and Aging at UCL. Anonymised data will be shared by request from bona fide investigators (https://skylark.ucl.ac.uk/NSHD/doku.php).

## Abbreviations

Aβ: β-amyloid
BaMoS: Bayesian Model Selection
DSST: Digit-symbol substitution test
FHS-CVS: Framingham heart study cardiovascular risk score
FLAIR: Fluid-attenuated inversion recovery
FNAME-12: Face-name test
MCI: Mild cognitive impairment
MMSE: Mini-mental state examination
MRI: Magnetic resonance imaging
NSHD: National Survey of Health and Development
PACC: Preclinical Alzheimer’s cognitive composite
PET: Positron emission tomography
SEP: Socioeconomic position
TIV: Total intracranial volume
UPDRS: United Parkinson’s disease rating scale
WMHV: White matter hyperintensity volume

## References

[1] Lane CA, Parker TD, Cash DM, et al. Study protocol: Insight 46 - a neuroscience sub-study of the MRC National Survey of Health and Development. BMC Neurol. 2017;17(1). 10.1186/s12883-017-0846-x 10.1186/s12883-017-0846-x PMC5395844 28420323

[2] Murray-Smith H, Barker S, Barkhof F, et al. Updating the study protocol: insight 46– a longitudinal neuroscience sub-study of the MRC National Survey of Health and Development– phases 2 and 3. BMC Neurol. 2024;24(1):40. 10.1186/s12883-023-03465-3 38263061 10.1186/s12883-023-03465-3 PMC10804658

[3] James SN, Lane CA, Parker TD, et al. Using a birth cohort to study brain health and preclinical dementia: recruitment and participation rates in insight 46. BMC Res Notes. 2018;11(1):885. 10.1186/s13104-018-3995-0 30545411 10.1186/s13104-018-3995-0 PMC6293512

[4] Pigeon D. Tests used in the 1954 and 1957 surveys. The home and the school. Edited by Douglas, J. Macgibbon & Key; 1964.

[5] Pierce MB, Silverwood RJ, Nitsch D, et al. Clinical disorders in a post war British cohort reaching retirement: evidence from the first National birth cohort study. Bayer A. Ed PLoS One. 2012;7(9):e44857. 10.1371/journal.pone.0044857 10.1371/journal.pone.0044857 PMC3447001 23028647

[6] Lane CA, Barnes J, Nicholas JM, et al. Associations between vascular risk across adulthood and brain pathology in late life: evidence from a British birth cohort. JAMA Neurol. 2020;77(2):175–83. 10.1001/jamaneurol.2019.3774 31682678 10.1001/jamaneurol.2019.3774 PMC6830432

[7] Rawle MJ, Davis D, Bendayan R, Wong A, Kuh D, Richards M. Apolipoprotein-E (Apoe) ϵ4 and cognitive decline over the adult life course. Transl Psychiatry. 2018;8(1):18. 10.1038/s41398-017-0064-8 29317609 10.1038/s41398-017-0064-8 PMC5802532

[8] Goetz CG, Tilley BC, Shaftman SR, et al. Movement disorder society-sponsored revision of the unified parkinson’s disease rating scale (MDS-UPDRS): scale presentation and clinimetric testing results. Mov Disord. 2008;23(15):2129–70. 10.1002/mds.22340 19025984 10.1002/mds.22340

[9] Devenney E, Hodges JR. The mini-mental state examination: pitfalls and limitations. Pract Neurol. 2017;17(1):79–80. 10.1136/practneurol-2016-001520 27903765 10.1136/practneurol-2016-001520

[10] Jaeger J. Digit symbol substitution test. J Clin Psychopharmacol. 2018;38(5):513–9. 10.1097/JCP.0000000000000941 30124583 10.1097/JCP.0000000000000941 PMC6291255

[11] Wechsler D. Wechsler memory scale-revised. *Psychol Corp*. Published online. 1987.

[12] Papp KV, Amariglio RE, Dekhtyar M, et al. Development of a psychometrically equivalent short form of the face-name associative memory exam for use along the early alzheimers disease trajectory. Clin Neuropsychol. 2014;28(5):771–85. 10.1080/13854046.2014.911351 24815535 10.1080/13854046.2014.911351 PMC4134419

[13] Lu K, Nicholas JM, Collins JD, et al. Cognition at age 70: life course predictors and associations with brain pathologies. Neurology. 2019;93(23):E2144–56. 10.1212/WNL.0000000000008534 31666352 10.1212/WNL.0000000000008534 PMC6937487

[14] Donohue MC, Sperling RA, Salmon DP, et al. The preclinical alzheimer cognitive composite. JAMA Neurol. 2014;71(8):961. 10.1001/jamaneurol.2014.803 24886908 10.1001/jamaneurol.2014.803 PMC4439182

[15] Rami L, Mollica MA, Garcfa-Sanchez C, et al. The subjective cognitive decline questionnaire (SCD-Q): a validation study. J Alzheimer’s Dis. 2014;41(2):453–66. 10.3233/JAD-132027 24625794 10.3233/JAD-132027

[16] Galvin JE, Roe CM, Powlishta KK, et al. The AD8: a brief informant interview to detect dementia. Neurology. 2005;65(4):559–64. 10.1212/01.wnl.0000172958.95282.2a 16116116 10.1212/01.wnl.0000172958.95282.2a

[17] Spielberger CD, Gorsuch RL, Lushene RE, Vagg PR, Jacobs GA. Manual for the state-trait anxiety inventory. Consult Psychol. Published online. 1983.

[18] Parker TD, Cash DM, Lane CA, et al. Amyloid β influences the relationship between cortical thickness and vascular load. Alzheimer’s Dement Diagnosis Assess Dis Monit. 2020;12(1). 10.1002/dad2.12022 10.1002/dad2.12022 PMC7163924 32313829

[19] Sudre CH, Cardoso MJ, Bouvy WH, Biessels GJ, Barnes J, Ourselin S. Bayesian model selection for pathological neuroimaging data applied to white matter lesion segmentation. IEEE Trans Med Imaging. 2015;34(10):2079–102. 10.1109/TMI.2015.2419072 25850086 10.1109/TMI.2015.2419072

[20] Leung KK, Barnes J, Modat M, et al. Brain MAPS: an automated, accurate and robust brain extraction technique using a template library. NeuroImage. 2011;55(3):1091–108. 10.1016/j.neuroimage.2010.12.067 21195780 10.1016/j.neuroimage.2010.12.067 PMC3554789

[21] Cardoso MJ, Leung K, Modat M, et al. STEPS: similarity and truth estimation for propagated segmentations and its application to hippocampal segmentation and brain parcelation. Med Image Anal. 2013;17(6):671–84. 10.1016/j.media.2013.02.006 23510558 10.1016/j.media.2013.02.006

[22] Malone IB, Leung KK, Clegg S, et al. Accurate automatic estimation of total intracranial volume: a nuisance variable with less nuisance. NeuroImage. 2015;104:366–72. 10.1016/j.neuroimage.2014.09.034 25255942 10.1016/j.neuroimage.2014.09.034 PMC4265726

[23] Chatfield MD, Brayne CE, Matthews FE. A systematic literature review of attrition between waves in longitudinal studies in the elderly shows a consistent pattern of dropout between differing studies. J Clin Epidemiol. 2005;58(1):13–9. 10.1016/j.jclinepi.2004.05.006 15649666 10.1016/j.jclinepi.2004.05.006

[24] Kuh D, Wong A, Shah I, et al. The MRC National Survey of Health and Development reaches age 70: maintaining participation at older ages in a birth cohort study. Eur J Epidemiol. 2016;31(11):1135–47. 10.1007/s10654-016-0217-8 27995394 10.1007/s10654-016-0217-8 PMC5206260

[25] Pavisic I, Lu K, Keuss SE, et al. Subjective cognitive complaints at age 70: associations with amyloid and mental health. JNNP. 2021;92(11):1215–21. 10.1136/jnnp-202-325620 10.1136/jnnp-2020-325620 PMC8522456 34035132

[26] Mihelic AH, Crimmins EM. Loss to follow-up in a sample of Americans 70 years of age and older: the LSOA 1984–1990. Journals Gerontol Ser B Psychol Sci Soc Sci. 1997;52B(1):S37–48. 10.1093/geronb/52B.1.S37 10.1093/geronb/52b.1.s37 9008680

[27] Grill JD, Kwon J, Teylan MA, et al. Retention of alzheimer disease research participants. Alzheimer Dis Assoc Disord. 2019;33(4):299–306. 10.1097/WAD.0000000000000353 31567302 10.1097/WAD.0000000000000353 PMC6878201

[28] Gavett BE, Gurnani AS, Saurman JL et al. Practice effects on story memory and list learning tests in the neuropsychological assessment of older adults. Ito E, ed. PLoS One. 2016;11(10):e0164492. 10.1371/journal.pone.0164492 10.1371/journal.pone.0164492 PMC5053775 27711147

[29] Samaroo A, Amariglio RE, Burnham S, et al. Diminished learning over repeated exposures (LORE) in preclinical alzheimer’s disease. Alzheimer’s Dement Diagnosis Assess Dis Monit. 2020;12(1). 10.1002/dad2.12132 10.1002/dad2.12132 PMC7784542 33426266

[30] Weintraub S, Salmon D, Mercaldo N, et al. The alzheimer’s disease centers’ uniform data set (UDS): the neuropsychological test battery. Alzheimer Dis Assoc Disorder. 2009;23(2):91–101. 10.1097/WAD.0b013e318191c7dd 10.1097/WAD.0b013e318191c7dd PMC2743984 19474567

[31] Salthouse TA. Trajectories of normal cognitive aging. Psychol Aging. 2019;34(1):17–24. 10.1037/pag0000288 30211596 10.1037/pag0000288 PMC6367038

[32] Mormino EC, Papp KV, Rentz DM, et al. Early and late change on the preclinical alzheimer’s cognitive composite in clinically normal older individuals with elevated amyloid. Alzheimers Dement. 2017;13(9):1004–12. 10.1016/j.jalz.2017.01.018 28253478 10.1016/j.jalz.2017.01.018 PMC5573651

