# Supplementary material for: Retention and longitudinal change in Insight 46, an intensive neuroscience sub-study of the 1946 British birth cohort
Source: BMC Res Notes. 2026 Jan 22;19:41. doi: 10.1186/s13104-025-07323-y (PMC12837026; doi:10.1186/s13104-025-07323-y)
Supplement: Supplementary file 1 — Supplementary Material 1 [file 13104_2025_7323_MOESM1_ESM.doc]

Table S1. Independent associations of measures with participant retention (max. n=462)

| **Measure** | **Returners**  **(max. n=413)** | **Non-returners**  **(max. n=49)** | **Returner vs non-returner status: unadjusted results** | | | **Returner vs non-returner status: adjusted results†** | | |
| --- | --- | --- | --- | --- | --- | --- | --- | --- |
|  |  |  | **OR** | **95% CI** | **p-value** | **OR** | **95% CI** | **p-value** |
| Age at baseline Insight 46 visit, years, mean (SD) | 70.6 (0.7) | 71.1 (0.6) | 0.28 | 0.16, 0.50 | <0.01* | 0.30 | 0.17, 0.53 | <0.01* |
| Female sex (versus male), n (%) | 196 (47.5) | 31 (63.3) | 0.49 | 0.25, 0.93 | 0.03* | 0.41 | 0.21, 0.81 | 0.01* |
| PACC, z-score, mean (SD) | 0.01 (0.73) | -0.13 (0.80) | 1.28 | 0.86, 1.90 | 0.22 | 1.50 | 0.97, 2.33 | 0.07 |
| Aβ+ PET status (Aβ- as reference), n (%) | 92 (22.3) | 18 (36.7) | 0.48 | 0.24, 0.93 | 0.03* | 0.52 | 0.26, 1.03 | 0.06 |

Abbreviations: PACC = preclinical Alzheimer cognitive composite; Aβ = β-amyloid; PET = positron emission tomography. Measures were assessed as predictors in a single model in order to determine their independent effects. †adjusted for age at baseline Insight 46 visit, sex, childhood cognition, education, socioeconomic position. *significant at p≤0.05

Table S2. Independent associations of measures with participant retention (max. n=502)

| **Measure** | **Returners**  **(max. n=442)** | **Non-returners**  **(max. n=60)** | **Returner vs non-returner status: unadjusted results** | | | **Returner vs non-returner status: adjusted results†** | | |
| --- | --- | --- | --- | --- | --- | --- | --- | --- |
|  |  |  | **OR** | **95% CI** | **p-value** | **OR** | **95% CI** | **p-value** |
| Age at baseline Insight 46 visit, years, mean (SD) | 70.6 (0.7) | 71.1 (0.6) | 0.31 | 0.19, 0.51 | <0.01* | 0.33 | 0.20, 0.54 | <0.01* |
| Female sex (versus male), n (%) | 209 (47.3) | 37 (61.7) | 0.48 | 0.26, 0.87 | 0.02* | 0.41 | 0.22, 0.77 | <0.01* |
| PACC, z-score, mean (SD) | 0.02 (0.72) | -0.13 (0.79) | 1.46 | 1.01, 2.10 | 0.04* | 1.75 | 1.16, 2.64 | <0.01* |
| Completion of neuroimaging (versus none), n (%) | 421 (92.3) | 50 (83.3) | 4.22 | 1.79, 9.98 | <0.01* | 4.42 | 1.83, 10.66 | <0.01* |

Abbreviations: PACC = preclinical Alzheimer cognitive composite. Measures were assessed as predictors in a single model in order to determine their independent effects. †adjusted for age at baseline Insight 46 visit, sex, childhood cognition, education, socioeconomic position. *significant at p≤0.05

Table S3. Sensitivity analysis excluding participants classified as having dementia or mild cognitive impairment (max. n=488)

| **Measure** | | **Returners**  **(max. n=432)** | **Non-returners**  **(max. n=56)** | **Returner vs non-returner status: unadjusted results** | | | **Returner vs non-returner status: adjusted results†** | | | | |
| --- | --- | --- | --- | --- | --- | --- | --- | --- | --- | --- | --- |
|  |  |  |  | **OR** | **95% CI** | **p** | **OR** | | **95% CI** | **p** | |
| **Demographic and life-course** | |  | | | | | | | | | |
| Age at baseline Insight 46 visit, years, mean (SD) | | 70.6 (0.7) | 71.1 (0.6) | 0.31 | 0.19, 0.52 | <0.01* | 0.33 | 0.19, 0.54 | | | <0.01* |
| Female sex (versus male), n (%) | | 205 (47.5) | 34 (60.7) | 0.58 | 0.33, 1.03 | 0.06 | 0.52 | 0.29, 0.94 | | | 0.03* |
| Education  level | Advanced (versus none), n (%)  Ordinary (versus none), n (%) | 228 (52.8)  136 (31.5) | 39 (69.6)  10 (17.9) | 0.60  1.40 | 0.26, 1.41  0.51, 3.84 | 0.06 | 0.56  1.31 | 0.20, 1.55  0.44, 3.88 | | | 0.09 |
| SEP, non-manual job (versus manual), n (%) | | 368 (85.2) | 49 (87.5) | 0.82 | 0.36, 1.89 | 0.64 | 0.92 | 0.37, 2.31 | | | 0.86 |
| Childhood cognition, z-score, mean (SD) | | 0.39 (0.73) | 0.47 (0.82) | 0.87 | 0.59, 1.27 | 0.46 | 1.10 | 0.70, 1.74 | | | 0.68 |
| APOE ε4 carrier (non-carrier as reference), n (%) | | 127 (29.5)  n=430^a^ | 17 (30.4) | 0.96 | 0.52, 1.76 | 0.90 | 0.86 | 0.45, 1.62 | | | 0.64 |
| Disease burden at age 69 | ≥3 (versus none), n (%)  2 (versus none), n (%)  1 (versus none), n (%) | 64 (15.0)  88 (20.6)  159 (37.2)  n=427^a^ | 9 (17.0)  10 (18.9)  19 (35.9)  n=53^a^ | 0.92  1.14  1.08 | 0.38, 2.22  0.49, 2.65  0.53, 2.22 | 0.97 | 0.88  1.12  0.98 | 0.35, 2.18  0.47, 2.70  0.46, 2.08 | | | 0.97 |
| Self-rated health at age 68 | Excellent (versus fair/poor), n (%)  Very good (versus fair/poor), n (%)  Good (versus fair/poor), n (%) | 61 (14.5)  213 (50.7)  114 (27.1)  n=420^a^ | 4 (7.8)  27 (52.9)  17 (33.3)  n=51^a^ | 1.43  0.74  0.63 | 0.30, 6.78  0.21, 2.58  0.17, 2.28 | 0.52 | 1.79  0.96  0.85 | 0.36, 8.92  0.26, 2.49  0.22, 3.22 | | | 0.67 |
| FHS Cardiovascular Risk Score age 69 | High (versus low), n (%)  Intermediate (versus low), n (%) | 255 (60.3)  125 (29.6)  n=423^a^ | 31 (60.8)  14 (27.5)  n=51^a^ | 1.15  1.25 | 0.45, 2.91  0.45, 3.45 | 0.91 | 0.60  1.38 | 0.19, 1.88  0.47, 4.05 | | | 0.19 |
| **Baseline Insight 46** | |  | | | | | | | | | |
| UPDRS, score out of 52, median (IQR) | | 3 (1-6) | 4 (2-6) | 0.96 | 0.90, 1.03 | 0.23 | 0.98 | 0.91, 1.05 | | | 0.50 |
| Informant concern, AD8 ≥2 (<2 as reference), n (%) | | 16 (3.7) | 2 (3.6) | 1.04 | 0.23, 4.64 | 0.96 | 0.93 | 0.19, 4.45 | | | 0.93 |
| Major brain disorder (none as reference), n (%) | | 32 (7.4) | 3 (5.4) | 1.41 | 0.42, 4.78 | 0.58 | 1.29 | 0.37, 4.49 | | | 0.69 |
| Subjective decline, MyCog score out of 20, median (IQR) | | 4 (2-7) | 3 (1-5) | 1.03 | 0.95, 1.11 | 0.46 | 1.03 | 0.95, 1.11 | | | 0.51 |
| Anxiety levels | State score out of 80, median (IQR) | 28 (23-34) | 30 (25-39) | 0.97 | 0.94, 1.01 | 0.10 | 0.98 | 0.95, 1.02 | | | 0.32 |
|  | Traits score out of 80, median (IQR) | 30 (26-26) | 32 (26-40) | 0.98 | 0.95, 1.01 | 0.20 | 0.98 | 0.95, 1.02 | | | 0.40 |
| MMSE, score out of 30, median (IQR) | | 29 (29-30) | 29 (29-30) | 0.98 | 0.71, 1.35 | 0.91 | 1.06 | 0.75, 1.49 | | | 0.74 |
| DSST, score out of 93, mean (SD) | | 48.1 (10.2) | 46.9 (9.9) | 1.01 | 0.98, 1.04 | 0.39 | 1.03 | 0.99, 1.06 | | | 0.11 |
| LMDR, score out of 25, mean (SD) | | 11.7 (3.6) | 11.0 (3.3) | 1.06 | 0.98, 1.14 | 0.16 | 1.12 | 1.02, 1.23 | | | 0.01* |
| Face-name test, score out of 96, mean (SD) | | 66.2 (17.5)  n=430^a^ | 64.8 (18.0) | 1.00 | 0.99, 1.02 | 0.56 | 1.02 | 1.00, 1.04 | | | 0.07 |
| PACC, z-score, mean (SD) | | 0.05 (0.67) | -0.04 (0.66) | 1.23 | 0.82, 1.86 | 0.32 | 1.85 | 1.12, 3.08 | | | 0.02* |
| Completion of neuroimaging (none as reference), n (%) | | 411 (95.1) | 47 (83.9) | 3.75 | 1.62, 8.66 | <0.01* | 3.70 | 1.58, 8.66 | | | <0.01* |
| Aβ+ PET status (Aβ- as reference), n (%) | | 87 (21.6)  n=403^a^ | 17 (37.0)  n=46^a^ | 0.47 | 0.25, 0.89 | 0.02* | 0.42 | 0.21, 0.83 | | | 0.01* |
| WMHV on MRI, ml, median (IQR) | | 3.1 (1.6-6.8)  n=398^a^ | 3.0 (1.7-5.8)  n=45^a^ | 1.00 | 0.95, 1.06 | 0.90 | 1.02 | 0.96, 1.09 | | | 0.57 |
| Whole brain volume on MRI, ml, mean (SD) | | 1105 (96)  n=408^a^ | 1073 (105)  n=47^a^ | 1.01 | 1.00, 1.01 | 0.04* | 1.01 | 1.00, 1.01 | | | 0.09 |
| Total hippocampal volume on MRI, ml, mean (SD) | | 6.29 (0.66)  n=408^a^ | 6.19 (0.68)  n=47^a^ | 1.12 | 0.66, 1.91 | 0.67 | 1.00 | 0.58, 1.73 | | | 1.00 |

Abbreviations: SEP = socioeconomic position; UPDRS = unified Parkinson’s disease rating scale; MCI = mild cognitive impairment; MMSE = mini-mental state examination; DSST = digit symbol substitution test; LMDR = logical memory delayed recall; PACC = preclinical Alzheimer cognitive composite; APOE = apolipoprotein E; Aβ = β-amyloid; PET = positron emission tomography; WMHV = white matter hyperintensity volume; MRI = magnetic resonance imaging. †adjusted for age at baseline Insight 46 visit, sex, childhood cognition, education, socioeconomic position. *significant at p≤0.05. ^a^number of participants with available data if below maximum possible.

Table S4. Sensitivity analysis excluding participants who did not return for follow-up because they were deceased (max. n=488)

| **Measure** | | **Returners**  **(max. n=442)** | **Non-returners**  **(max. n=46)** | **Returner vs non-returner status: unadjusted results** | | | **Returner vs non-returner status: adjusted results†** | | | | |
| --- | --- | --- | --- | --- | --- | --- | --- | --- | --- | --- | --- |
|  |  |  |  | **OR** | **95% CI** | **p** | **OR** | | **95% CI** | **p** | |
| **Demographic and life-course** | |  | | | | | | | | | |
| Age at baseline Insight 46 visit, years, mean (SD) | | 70.6 (0.7) | 71.1 (0.6) | 0.27 | 0.15, 0.47 | <0.01* | 0.28 | 0.16, 0.49 | | | <0.01* |
| Female sex (versus male), n (%) | | 209 (47.3) | 25 (54.4) | 0.75 | 0.41, 1.39 | 0.36 | 0.66 | 0.35, 1.25 | | | 0.20 |
| Education  level | Advanced (versus none), n (%)  Ordinary (versus none), n (%) | 233 (52.7)  140 (31.7) | 32 (69.6)  8 (17.4) | 0.63  1.52 | 0.25, 1.58  0.51, 4.56 | 0.08 | 0.61  1.40 | 0.21, 1.81  0.44, 4.51 | | | 0.16 |
| SEP, non-manual job (versus manual), n (%) | | 374 (84.6) | 41 (89.1) | 0.67 | 0.26, 1.76 | 0.42 | 0.72 | 0.25, 2.06 | | | 0.54 |
| Childhood cognition, z-score, mean (SD) | | 0.38 (0.73) | 0.43 (0.78) | 0.91 | 0.60, 1.38 | 0.67 | 1.14 | 0.69, 1.88 | | | 0.60 |
| APOE ε4 carrier (non-carrier as reference), n (%) | | 129 (29.3)  n=440^a^ | 13 (28.3) | 1.05 | 0.54, 2.07 | 0.88 | 0.95 | 0.47, 1.94 | | | 0.90 |
| Disease burden at age 69 | ≥3 (versus none), n (%)  2 (versus none), n (%)  1 (versus none), n (%) | 67 (15.2)  89 (20.4)  163 (37.3)  n=437^a^ | 4 (9.1)  7 (15.9)  18 (40.9)  n=44^a^ | 2.13  1.62  1.15 | 0.68, 6.68  0.63, 4.13  0.56, 2.38 | 0.52 | 2.03  1.61  1.05 | 0.62, 6.58  0.61, 4.23  0.49, 2.26 | | | 0.54 |
| Self-rated health at age 68 | Excellent (versus fair/poor), n (%)  Very good (versus fair/poor), n (%)  Good (versus fair/poor), n (%) | 61 (14.2)  217 (50.6)  118 (27.5)  n=429^a^ | 4 (9.5)  24 (57.1)  12 (28.6)  n=42^a^ | 0.92  0.55  0.60 | 0.16, 5.32  0.12, 2.43  0.13, 2.80 | 0.71 | 1.29  0.77  0.89 | 0.21, 7.94  0.17, 3.60  0.18, 4.38 | | | 0.83 |
| FHS Cardiovascular Risk Score age 69 | High (versus low), n (%)  Intermediate (versus low), n (%) | 261 (60.3)  129 (29.8)  n=433^a^ | 26 (60.5)  12 (27.9)  n=43^a^ | 1.25  1.17 | 0.42, 3.75  0.43, 3.20 | 0.92 | 0.88  1.40 | 0.24, 3.21  0.44, 4.45 | | | 0.63 |
| **Baseline Insight 46** | |  | | | | | | | | | |
| UPDRS, score out of 52, median (IQR) | | 3 (1-6) | 3 (1-5)  n=45^a^ | 1.02 | 0.94, 1.10 | 0.69 | 1.05 | 0.95, 1.15 | | | 0.33 |
| Informant concern, AD8 ≥2 (<2 as reference), n (%) | | 23 (5.2) | 3 (6.5) | 0.79 | 0.23, 2.73 | 0.71 | 0.83 | 0.23, 3.03 | | | 0.78 |
| Dementia or MCI (none as reference), n (%) | | 10 (2.3) | 4 (8.7) | 0.24 | 0.07, 0.81 | 0.02* | 0.25 | 0.07, 0.95 | | | 0.04* |
| Major brain disorder (none as reference), n (%) | | 38 (8.6) | 3 (6.5) | 1.35 | 0.40, 4.55 | 0.63 | 1.20 | 0.34, 4.18 | | | 0.78 |
| Subjective decline, MyCog score out of 20, median (IQR) | | 4 (2-7) | 3 (0-6) | 1.01 | 0.93, 1.10 | 0.78 | 1.01 | 0.93, 1.10 | | | 0.74 |
| Anxiety levels | State score out of 80, median (IQR) | 28 (23-34) | 30 (25-40) | 0.97 | 0.93, 1.00 | 0.08 | 0.98 | 0.94, 1.01 | | | 0.22 |
|  | Traits score out of 80, median (IQR) | 30 (26-36) | 32 (26-39) | 0.99 | 0.95, 1.03 | 0.64 | 1.00 | 0.96, 1.04 | | | 0.88 |
| MMSE, score out of 30, median (IQR) | | 30 (29-30) | 29 (29-30) | 1.23 | 0.96, 1.59 | 0.10 | 1.26 | 0.96, 1.65 | | | 0.10 |
| DSST, score out of 93, mean (SD) | | 47.8 (10.4)  n=441^a^ | 46.2 (10.8) | 1.02 | 0.99, 1.05 | 0.31 | 1.02 | 0.99, 1.06 | | | 0.15 |
| LMDR, score out of 25, mean (SD) | | 11.6 (3.7) | 10.4 (3.9) | 1.09 | 1.01, 1.18 | 0.03* | 1.15 | 1.05, 1.26 | | | <0.01* |
| Face-name test, score out of 96, mean (SD) | | 65.5 (18.1)  n=440^a^ | 60.3 (21.0) | 1.01 | 1.00, 1.03 | 0.07 | 1.03 | 1.01, 1.05 | | | <0.01* |
| PACC, z-score, mean (SD) | | 0.02 (0.72) | -0.24 (0.85) | 1.52 | 1.05, 2.22 | 0.03* | 1.96 | 1.26, 3.05 | | | <0.01* |
| Completion of neuroimaging (none as reference), n (%) | | 421 (95.3) | 38 (82.6) | 4.22 | 1.75, 10.17 | <0.01* | 4.17 | 1.71, 10.17 | | | <0.01* |
| Aβ+ PET status (Aβ- as reference), n (%) | | 92 (22.3)  n=413^a^ | 14 (37.8)  n=37^a^ | 0.47 | 0.23, 0.95 | 0.04* | 0.43 | 0.20, 0.91 | | | 0.03* |
| WMHV on MRI, ml, median (IQR) | | 3.1 (1.6-6.8)  n=408^a^ | 3.3 (1.7-5.8)  n=35^a^ | 0.99 | 0.92, 1.06 | 0.84 | 1.01 | 0.94, 1.09 | | | 0.71 |
| Whole brain volume on MRI, ml, mean (SD) | | 1104 (98)  n=418^a^ | 1087 (98)  n=38^a^ | 1.00 | 1.00, 1.01 | 0.20 | 1.00 | 1.00, 1.01 | | | 0.44 |
| Total hippocampal volume on MRI, ml, mean (SD) | | 6.28 (0.67)  n=418^a^ | 6.18 (0.76)  n=38^a^ | 1.27 | 0.72, 2.24 | 0.42 | 1.13 | 0.64, 2.02 | | | 0.67 |

Abbreviations: SEP = socioeconomic position; UPDRS = unified Parkinson’s disease rating scale; MCI = mild cognitive impairment; MMSE = mini-mental state examination; DSST = digit symbol substitution test; LMDR = logical memory delayed recall; PACC = preclinical Alzheimer cognitive composite; APOE = apolipoprotein E; Aβ = β-amyloid; PET = positron emission tomography; WMHV = white matter hyperintensity volume; MRI = magnetic resonance imaging. †adjusted for age at baseline Insight 46 visit, sex, childhood cognition, education, socioeconomic position. *significant at p≤0.05. ^a^number of participants with available data if below maximum possible.

Table S5. Sensitivity analysis: excluding participants who completed a remote assessment at follow-up (max. n=413)

| **Measure** | | **Baseline** | **Follow-up** | **Percentage point or mean (SD) change** | **p-value** |
| --- | --- | --- | --- | --- | --- |
| UPDRS, score out of 52, median (IQR) | | 3 (1-6) | 6 (3-8) | 2.66 (4.42) | <0.01* |
| Anxiety levels | State score out of 80, median (IQR) | 28 (23-34) | 27 (23-33)  n=412^a^ | -0.88 (6.65)  n=412^a^ | 0.02 |
|  | Trait score out of 80, median (IQR) | 30 (26-36) | 30 (26-36)  n=412^a^ | 0.02 (5.48)  n=412^a^ | 0.82 |
| Informant concern, AD8 ≥2, n (%) | | 19 (4.6) | 72 (17.4) | 12.8 | <0.01* |
| Dementia or MCI, n (%) | | 8 (1.9) | 24 (5.8) | 3.9 | <0.01* |
| Major brain disorder, n (%) | | 36 (8.7) | 40 (9.7) | 1.0 | 0.13 |
| Subjective decline, MyCog score out of 20, median (IQR) | | 4 (1-7) | 4 (1-8) | 0.45 (3.10) | <0.01* |
| DSST, score out of 93, mean (SD) | | 47.9 (10.3)  n=412^a^ | 46.1 (10.9)  n=412^a^ | -1.80 (5.21)  n=412^a^ | <0.01* |
| LMDR, score out of 25, mean (SD) | | 11.5 (3.7) | 12.2 (3.7) | 0.64 (3.17) | <0.01* |
| Face-name test, score out of 96, mean (SD) | | 65.4 (17.9)  n=411^a^ | 66.1 (19.5)  n=412^a^ | 0.85 (10.33)  n=410^a^ | 0.04 |
| PACC, z-score, mean (SD) | | 0.02 (0.71) | -0.05 (0.82) | -0.07 (0.44) | <0.01* |

Abbreviations: UPDRS = unified Parkinson’s disease rating scale; MCI = mild cognitive impairment; DSST = digit symbol substitution test; LMDR = logical memory delayed recall; PACC = preclinical Alzheimer cognitive composite.

*significant at p≤0.05 ^a^number of participants with available data if below maximum possible.

NB. MMSE, body mass index & blood pressure were not assessed remotely so are omitted from this analysis.

Table S6. Post-hoc analysis: changes in cognitive measures across whole sample and by Aβ status, unweighted and weighted (max. n=413)

| **Measure** | **Unweighted** | | | | **Weighted** | | | |
| --- | --- | --- | --- | --- | --- | --- | --- | --- |
|  | **Mean change across whole sample** | **Difference in rate of change between Aβ positive and negative participants** | | | **Mean change across whole sample** | **Difference in rate of change between Aβ positive and negative participants** | | |
|  |  | **β** | **95% CI** | **p-value** |  | **β** | **95% CI** | **p-value** |
| Subjective cognition, MyCog | 0.41 | 0.07 | -0.23, 0.36 | 0.65 | 0.43 | 0.08 | -0.18, 0.35 | 0.54 |
| MMSE | -0.31 | 0.01 | -0.12, 0.13 | 0.93 | -0.31 | -0.01 | -0.14, 0.13 | 0.92 |
| DSST | -1.93 | -0.11 | -0.62, 0.41 | 0.68 | -1.95 | -0.09 | -0.63, 0.45 | 0.74 |
| LMDR | 0.69 | 0.05 | -0.25, 0.35 | 0.76 | 0.70 | 0.04 | -0.24, 0.33 | 0.76 |
| Face-name test | 0.93 | -0.46 | -1.45, 0.53 | 0.36 | 0.88 | -0.53 | -1.72, 0.66 | 0.38 |
| PACC | -0.06 | -0.01 | -0.05, 0.03 | 0.68 | -0.06 | -0.01 | -0.07, 0.04 | 0.58 |

Abbreviations: MMSE = mini-mental state examination; DSST = digit symbol substitution test; LMDR = logical memory delayed recall; PACC = preclinical Alzheimer cognitive composite. Linear regression models were fitted to assess (a) changes in measures between baseline and follow-up and (b) whether rates of change in measures between baseline and follow-up differed by Aβ status. For (a) each model included change in the measure between baseline and follow-up as the outcome, and follow-up status as the predictor. For (b) each model included change in the measure between baseline and follow-up as the outcome, interval between baseline and follow-up in years as the explanatory variable, and interactions between interval and (i) baseline Aβ status and (ii) each covariate (age at baseline, sex, childhood cognition, education level and SEP). No constant term was included since the model estimates mean change over time. Models were additionally run using inverse probability weighting to account for any bias related to loss of participants to follow-up. Weights for probability of retention were estimated using a logistic regression model with follow-up status as the outcome, and sex, baseline Aβ status, age, and PACC as predictors.
